# CD56-negative NK cells with impaired effector function expand in CMV and EBV co-infected healthy donors with age

**DOI:** 10.18632/aging.101774

**Published:** 2019-01-27

**Authors:** Bojana Müller-Durovic, Jasmin Grählert, Oliver P. Devine, Arne N. Akbar, Christoph Hess

**Affiliations:** 1 University Hospital Basel, Department of Biomedicine, Basel, Switzerland; 2 Division of Infection and Immunity, University College London, London, UK; 3 Department of Medicine, University of Cambridge, Cambridge, UK; * Equal contribution

**Keywords:** aging, CMV, EBV, CD56-negative, NK cells, senescence, exhaustion

## Abstract

Natural killer cells lacking expression of CD56 (CD56^neg^ NK cells) have been described in chronic HIV and hepatitis C virus infection. Features and functions of CD56^neg^ NK cells in the context of latent infection with CMV and / or EBV with age are not known. In a cohort of healthy donors >60 years of age, we found that co-infection with CMV and EBV drives expansion of CD56^neg^ NK cells. Functionally, CD56^neg^ NK cells displayed reduced cytotoxic capacity and IFN-γ production, a feature that was enhanced with CMV / EBV co-infection. Further, the frequency of CD56^neg^ NK cells correlated with accumulation of end-stage-differentiated T cells and a reduced CD4 / CD8 T cell ratio, reflecting an immune risk profile. CD56^neg^ NK cells had a mature phenotype characterized by low CD57 and KIR expression and lacked characteristics of cell senescence. No changes in their activating NK cell receptor expression, and no upregulation of the negative co-stimulation receptors PD-1 or TIM-3 were observed. In all, our data identify expansion of dysfunctional CD56^neg^ NK cells in CMV^+^EBV^+^ elderly individuals suggesting that these cells may function as shape-shifters of cellular immunity and argue for a previously unrecognized role of EBV in mediating immune risk in the elderly.

## Introduction

CMV and EBV are the most ubiquitous herpes viruses, with a prevalence of up to 95% for EBV and close to 50% for CMV in the adult Western population [1]. Following primary infection, most often during early life, both viruses establish life-long latent infection. While immune-competent hosts are mostly asymptomatic, CMV and EBV can cause illness in immune-compromised individuals. Importantly, CMV is known to significantly shape the immune system with increasing age. Specifically, inflation of CMV-specific CD8^+^ T cells with a terminally differentiated phenotype (CD8^+^CD28^–^) and an inverted CD4 / CD8 T cell ratio have been described in CMV-positive individuals [2,3].

The OCTO Immune Longitudinal Study established an *immune risk profile* (IRP) – characterized by latent CMV infection, inversion of the CD4 / CD8 T cell ratio, and accumulation of T cells lacking expression of CD28 – which was predictive of 2-year mortality in healthy donors of more than 80 years of age [4,5]. Follow-up studies over the entire adult life span established that these immune changes as well as mortality rates associated with the IRP markedly increase in the age range of 60-94 years [6]. Recent work extended these findings, showing that CMV is a driving force behind the IRP [7]. The contribution of EBV to immune-senescence is far less well studied, not least because the high prevalence of EBV-positive individuals among the adult population is making detailed studies challenging.

NK cells are group 1 innate lymphoid cells (ILC-1) with high cytotoxic activity and an ability to produce large amounts of IFN-γ when interacting with infected or transformed target cells [8]. Human NK cells can be divided into two main populations based on their relative expression of the adhesion molecule CD56 and the low-affinity Fc receptor CD16 [9,10]. CD56^dim^ (CD56^+^CD16^++^) NK cells constitute the majority of NK cells in peripheral blood and represent the main effector population [9], while CD56^bright^ (CD56^++^CD16^–^) cells are predominantly found within lymphoid tissues and constitute 5-10% of peripheral blood NK cells [11]. Developmentally, CD56^bright^ NK cells are thought to be precursors of the more differentiated CD56^dim^ NK cell subset [12–14]. More recently, a third NK cell subset has been described that lacks CD56 expression (CD56^–^CD16^++^; referred to as CD56^neg^ NK cells throughout the manuscript) [15–21]. Loss of CD56 expression, in conjuncture with the lack of an alternative NK cell-specific marker in humans, complicates characterization of this NK cell subset. Earlier studies identified CD56^neg^ NK cells by exclusion of cells expressing CD3, CD4, CD14, and CD19 [19,22–24]. A more recent report further established exclusion of cells lacking expression of CD7 from the CD3-negative lymphocyte fraction as a more reliable means to exclude cells of the myeloid lineage (monocytes, dendritic cells) from the NK cell population [22,25,26].

Persistent viral infections have a significant impact on NK cell phenotype and function [27,28]. In chronic HIV infection, a dramatic increase in CD56^neg^ NK cells has been described [15–21]. Compared to CD56^dim^ NK cells these cells were shown to be markedly impaired in their capacity to secrete IFN-γ, lyse HLA-I-deficient target cells, and participate in antibody-dependent cytotoxicity (ADCC) [15,17,18,21,29]. Although less pronounced, expansion of CD56^neg^ NK cells was also reported in chronic hepatitis C virus (HCV) infection [23] and in patients with Burkitt’s lymphoma [30]. Similar to HIV-infected individuals, patients with chronic HCV infection accumulated CD56^neg^ NK cells that were impaired in their capacity to degranulate and secrete IFN-γ and TNF-α in response to target cell stimulation [23]. It has therefore been hypothesized that the expansion of this assumed defective CD56^neg^ NK cell population reflects a mechanism by which viruses subvert NK cell responses.

Here we performed phenotypic and functional analyses of CD56^neg^ NK cells in a cohort of healthy donors of >60 years of age (n=38, median 64 years, range 62-70 years) with known CMV and EBV serostatus. Specifically, we enumerated CD56^neg^ NK cells and tested their cytotoxic capacity in response to target cell and cytokine stimulation, determined the differentiation stage of CD56^neg^ NK cells relative to other NK cell subsets, and assessed cell senescence and exhaustion characteristics.

## RESULTS AND DISCUSSION

### CD56neg NK cells with impaired effector function expand in CMV / EBV co-infected hosts >60 years of age

**Figure 1:**
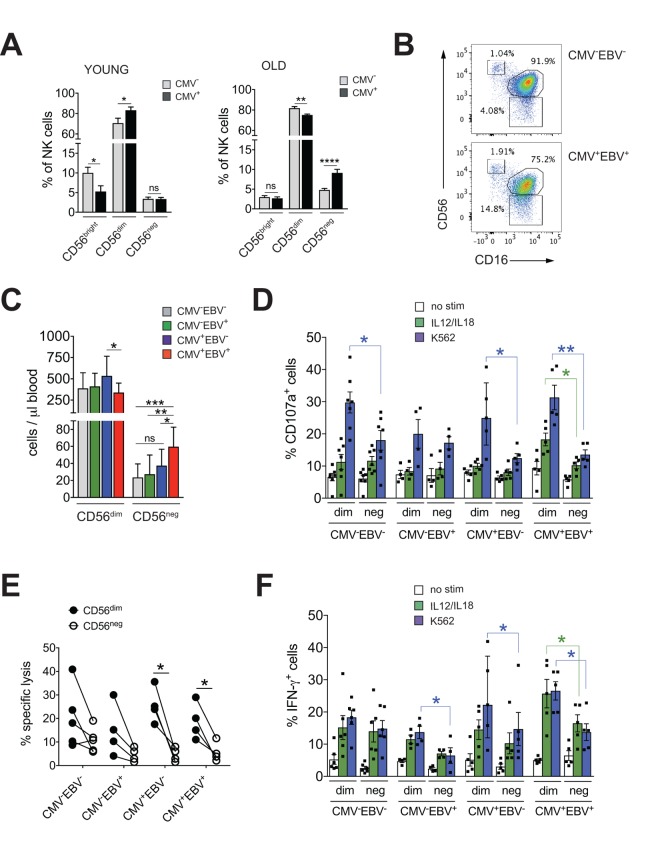
**CD56^neg^ NK cells with impaired effector function expand in CMV and EBV co-infected individuals >60 years of age.** (**A**) Frequencies of CD56^bright^, CD56^dim^ and CD56^neg^ NK cells in YOUNG (<35 years) CMV^–^ (gray bars, n=10/10) and CMV^+^ (black bars, n=10/10) individuals compared to OLD (>60 years) CMV^-^ (gray bars, n=20/21) and CMV^+^ (black bars, n=17/20) donors analyzed as in Supplementary Figure S1A. (**B**) Representative FACS dot plots from a CMV^–^EBV^–^ and a CMV^+^EBV^+^ donor are shown. Numbers indicate the percentage of cells within total NK cells in peripheral blood. (**C**) Absolute cell numbers for CD56^dim^ and CD56^neg^ NK cells – as determined by FACS analysis in total PBMCs– are shown in a cohort of HDs >60 years of age stratified as CMV^–^EBV^–^ (n=11/11), CMV^–^EBV^+^ (n=10/24), CMV^+^EBV^–^ (n=6/6), and CMV^+^EBV^+^ (n=12/14). (**D-F**) FACS-sorted CD56^dim^ and CD56^neg^ NK cells from CMV^–^EBV^–^ (n=7), CMV^–^EBV^+^ (n=4), CMV^+^EBV^-^ (n=4) and CMV^+^EBV^+^ (n=5) donors were either left un-stimulated (empty bars), stimulated with IL-12 / IL-18 (green bars) or K562 target cells (blue bars) and (**D**) CD107a expression (**E**) target cell lysis and (**F**) IFN-γ production were assessed after 6 hours of (co-)culture. Parametric data were compared by Student’s t-test and are shown as mean ± SEM, non-parametric data by Mann-Whitney test and are shown as median ± IQR, respectively. * p≤0.05, ** p≤0.005, *** p≤0.005.

The imprint of chronic viral infections on immunity is most pronounced during later stages of life. To study the impact of CMV infection on NK cell immunity during aging we first determined frequencies of NK cell subsets in 20 young (<35 years (median 31 years, range 25-34 years)) and 41 elderly (>60 years (median 64 years, range 62-70 years)) donors stratified according to CMV serostatus. Intriguingly, CMV infection in healthy donors >60 years of age was associated with a distinct increase in the frequency of CD56^neg^ and a decrease in CD56^dim^ NK cells (Figure 1A). In contrast, young CMV-positive donors had less CD56^bright^ NK cells but an increased proportion of CD56^dim^ cells (Figure 1A). No changes in the frequency of CD56^neg^ NK cells were seen in young CMV-positive donors (Figure 1A) – in line with 2 previous reports [31,32]. To delineate the relative contribution of CMV *vs*. EBV infection to the observed increase in CD56^neg^ NK cells, we next divided the cohort of elderly donors into CMV^–^EBV^–^ (n=11), EBV-positive (CMV^–^EBV^+^, n=24), CMV-positive (CMV^+^EBV^–^, n=6) and CMV^+^EBV^+^ (n=14) donors [33]. NK cells were identified in total PBMCs by gating on CD3^–^ and CD7^+^ positive lymphocytes [26], then divided into three subsets based on their CD56 and CD16 expression: CD56^bright^ (CD56^++^CD16^–^), CD56^dim^ (CD56^+^CD16^++^), and CD56^neg^ (CD56^–^CD16^++^) NK cells (Supplementary Figure 1A). When comparing NK cell subsets between cohort subgroups stratified according to CMV and EBV serostatus, we found a significant increase in frequency (Figure 1B, Supplementary Figure 1B) and absolute cell numbers (Figure 1C) of CD56^neg^ NK cells in CMV^+^EBV^+^ individuals only, although the low sample number in the CMV^+^EBV^–^ subgroup poses some limitation to this conclusion. Expansion of CD56^neg^ NK cells in CMV^+^EBV^+^ donors was accompanied by a reduction in CD56^dim^ NK cell numbers compared to CMV^+^ donors without EBV infection (Figure 1C).

CD56^neg^ NK cells with reduced cytotoxic function have been described in chronic HIV and HCV infection [15–21]. Aiming to investigate effector functions of CD56^neg^ NK cells in CMV and EBV co-infected donors, we FACS-sorted CD56^neg^ and CD56^dim^ NK cells from all 4 cohort subgroups stratified according to CMV and EBV serostatus as described above. We then assessed expression of CD107a and production of IFN-γ in response to stimulation with K562 target cells, IL-12 / IL-18 (Figure 1D, F) and the killing capacity toward K562 target cells (Figure 1E). We found a significant decrease in CD107a expression in CD56^neg^ NK cells compared to CD56^dim^ cells in CMV^–^EBV^–^ (n=7) and CMV^+^EBV^–^ (n=6) donors, and an even more pronounced decrease in CD107a in CD56neg NK cells from CMV^+^EBV^+^ (n=5) donors (Figure 1D). Moreover, in CMV^+^EBV^+^ individuals CD107a expression was decreased in response to *both* target cell and cytokine stimulation in the CD56^neg^ cell subset (Figure 1D). We then tested killing capacity of sorted CD56^dim^ and CD56^neg^ NK cells from all 4 cohort subgroups by measuring lysis of K562 target cells, a more direct measure of cytotoxic capacity. In co-culture with K562 target cells, CD56^neg^ NK cells from CMV^+^EBV^–^ and CMV^+^EBV^+^ donors had a significantly lower killing capacity than CD56^dim^ NK cells from the same donor (Figure 1E). Analogous to the expression of CD107a, production of IFN-γ was significantly lower in CD56^neg^ as compared to CD56^dim^ NK cells in CMV^–^EBV^+^(n=4), CMV^+^EBV^–^ (n=5) and CMV^+^EBV^+^ (n=5) donors after stimulation with K562 target cells (Figure 1F). In response to IL-12 / IL-18 stimulation, only CMV^+^EBV^+^ donors showed a significant reduction in IFN-γ production (Figure 1F). Intriguingly, CD56^dim^ NK cells from CMV-positive donors had a significantly higher capacity to secrete IFN-γ than those from CMV-negative individuals (Figure 1F). These data are in line with previous reports that established a role for CMV in shaping immune reactivity of CD56^dim^ NK cells both *in vitro* and *in vivo* [34,35], a phenomenon that was not recapitulated in the CD56^neg^ NK cell subset (Figure 1F). In all, our data suggested that CD56^neg^ NK cells had reduced cytotoxic capacity and IFN-γ production compared to CD56^dim^ NK cells, a feature that was further pronounced in the context of CMV / EBV co-infection.

### Frequencies of CD56^neg^ NK cells in donors >60 years of age correlate with the immune risk profile

**Figure 2:**
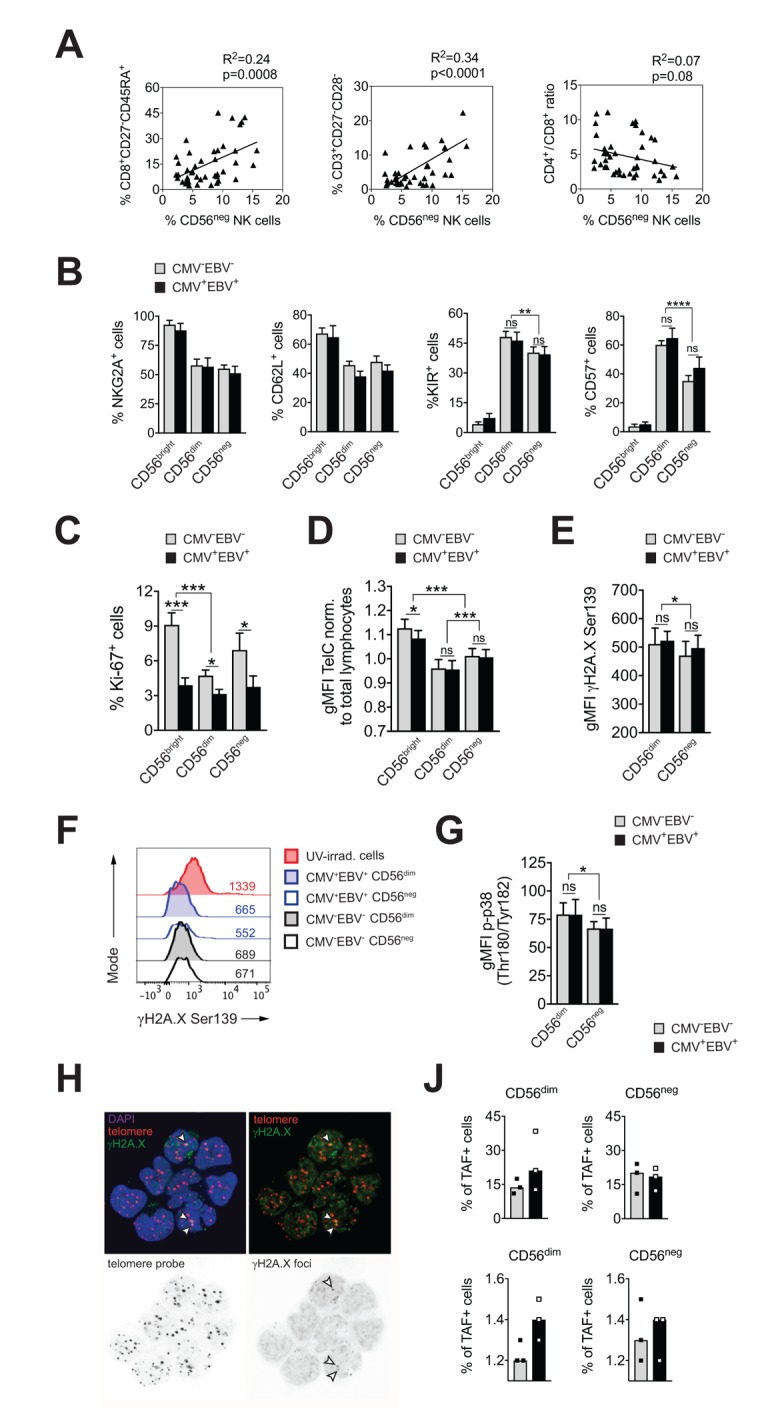
**CD56^neg^ NK cells do not acquire cell senescence characteristics.** (**A**) Frequencies of CD56^neg^ NK cells in relation to CD8^+^ EMRA T cells (left panel), CD27^-^CD28^-^ T cells (middle panel) and the CD4 / CD8 T cell ratio (right panel) as assessed by FACS analysis in total PBMCs (n=53/55). Data were analyzed by linear regression: correlation strength (R^2^) and statistical significance (p-value) are indicated for each scatter plot. (**B**) The differentiation stage of CD56^neg^ NK cells was assessed by FACS analysis for NKG2A, CD62L, KIR and CD57 expression in total PBMCs. CD56^neg^ NK cells were compared to CD56^bright^ and CD56^dim^ NK cells from CMV^–^EBV^–^ (gray bars, n=10/11) and CMV^+^EBV^+^ donors (black bars, n=10/14). (**C**) Proliferation of NK cell subsets from CMV^–^EBV^–^ (gray bars, n=10/11) and CMV^+^EBV^+^ (black bars, n=10/14) donors as assessed directly *ex vivo* by FACS analysis for Ki-67 expression. (**D**) Telomere length of NK cell subsets in CMV^–^EBV^–^ (gray bars, n=10/10) and CMV^+^EBV^+^ donors (black bars, n=10/14) as assessed by FACS-based FISH-technique. Data are shown as geometric mean of fluorescence intensity (gMFI) of the telomere probe (TelC), normalized to the gMFI TelC value of the total lymphocyte population for each donor. (**E**) Global phosphorylation of the histone H2A.X (γH2A.X Ser139) in CD56^dim^ and CD56^neg^ NK cells in CMV^–^EBV^–^ (gray bars, n=8/11) and CMV^+^EBV^+^ donors (black bars, n=9/14) as assessed directly *ex vivo* by FACS analysis. (**F**) Representative histograms for γH2A.X staining in a CMV^–^EBV^–^ (gray histograms) and CMV^+^EBV^+^ (blue histograms) donor. UV-irradiated PBMCs served as positive control. (**G**) Phosphorylation of p38-MAPK Thr180/Tyr182 in CD56^dim^ and CD56^neg^ NK cells in CMV^–^EBV^–^ (gray bars, n=8/11) and CMV^+^EBV^+^ (black bars, n=9/14) donors analyzed directly *ex vivo* by FACS analysis. (**H**) Representative telomere fluorescence in situ hybridization images showing overlay images of the nuclear staining (DAPI, purple) with telomere probe (red) and γH2A.X Ser139 (green) (top left) and co-localization of telomere probe and γH2A.X foci = telomere-associated fluorescence (TAF) (top right panel). White arrows indicate TAF. Greyscale stack images of the telomere probe (bottom left) and γH2A.X foci are shown (bottom right). (**J**) Cumulative data from CMV^–^EBV^–^ (gray bars, n=3) and CMV^+^EBV^+^ (black bars, n=3) donors are shown analyzed as in (**H**). Top panel shows the frequency of TAF+ cells, bottom panel the number of TAF / TAF+ cell in CD56^dim^ and CD56^neg^ NK cells. (**A**-**G**) Experiments were performed on total PBMCs. (**H, J**) Experiments were performed on FACS-sorted CD56^dim^ and CD56^neg^ NK cells. For parametric data mean ± SEM, for non-parametric data median ± IQR are shown. * p≤0.05, ** p≤0.005, *** p≤0.005, **** p≤0.0005, ns=not significant.

CMV infection has been associated with significant changes in T cell subset distribution with age. Population-based studies, pioneered by the Swedish longitudinal OCTO immune study, established an immune risk profile (IRP) characterized by CMV positivity, an inversed CD4 / CD8 T cell ratio and accumulation of end-differentiated T cells with poor proliferative capacity – an immunologic imprint that has been associated with a higher 2-year-mortaliity rate in healthy donors >60 years of age [6]. We next analyzed whether an increase in CD56^neg^ NK cells may be associated with the IRP. To this end, we performed phenotypic analysis of CD4^+^ and CD8^+^ T cells classified as naïve (N; CD27^+^, CD45RA^+^), central memory (CM; CD27^+^, CD45RA^–^), effector memory (EM; CD27^–^, CD45RA^–^) and terminally-differentiated effector memory (EMRA; CD27^–^, CD45RA^+^) T cells, and determined the number of double negative (DN; CD27^–^CD28^–^) T cells [36,37] in all 4 subgroups of the cohort (Supplementary Figure S1A). Indeed, frequencies of CD56^neg^ NK cells correlated with the percentage of end-differentiated T cells, specifically CD8^+^ EMRA and CD27^–^CD28^–^ T cells, and a reduced CD4 / CD8 T cell ratio (Figure 2A). When stratified to CMV and EBV serostatus, only donors that were either CMV- or EBV-positive (or both) showed a positive correlation between the frequency of CD56^neg^ NK and CD8^+^ EMRA T cells (Supplementary Figure S2A, left panel).

This newly identified association of CD56^neg^ NK cells with the IRP raised the question whether CD56^neg^ NK cells are a terminally-differentiated subset as well, and whether they display cell senescence characteristics. To determine the differentiation stage of CD56^neg^ NK cells we analyzed cell surface expression of NKG2A, CD62L, the Killer-cell Immunoglobulin-like Receptors (KIRs), and CD57. Expression of NKG2A and CD62L in NK cells is reciprocal to KIR and CD57 expression, with a step-wise reduction in NKG2A and CD62L and progressive acquisition of KIR and CD57 with differentiation [38,39]. In our cohort, CD56^neg^ NK cells displayed a decrease in NKG2A and CD62L expression and acquisition of KIR and CD57 compared to CD56^bright^ NK cells irrespective of the serostatus of the donors (Figure 2B and Supplementary Figure S2B), suggesting that CD56^neg^ cells have a mature phenotype. Compared to CD56^dim^ NK cells, however, CD56^neg^ NK cells expressed significantly less KIR and CD57 (Figure 2B and Supplementary Supplementary Figure S2B). CD57 expression defines mature NK cells with potent effector function [40], and acquisition of KIR is associated with ‘licensing’ of the NK cell (a process in which only cells that express KIR for self-MHC molecules acquire maximal functional capacity [41]). Absence of these two markers could point to either a more immature cell subset that has not acquired full effector functions, or a cell subset with faulty licensing, respectively, which would be in line with their reduced effector functions as shown in Figure 1D-F.

Having established that CD56^neg^ NK cells have a mature phenotype, we next investigated whether they acquire cell senescence characteristics. Loss of proliferative capacity is a hallmark of cell senescence. Therefore, we first assessed proliferation of NK cell subsets directly *ex vivo*, by staining for Ki-67, expression of which is found in cycling cells only. CD56^neg^ NK cells had intermediate levels of Ki-67 expression compared to CD56^bright^ and CD56^dim^ NK cells, recapitulating our phenotyping results in Figure 2B, that established an intermediate differentiation phenotype for the CD56^neg^ NK cell subset. Intriguingly, we observed reduced Ki-67 expression in all three NK cell subsets in CMV^+^EBV^+^ donors when compared to CMV^–^EBV^–^ individuals and this reduction was most pronounced in CD56^bright^ NK cells (Figure 2C). There was no significant difference in Ki-67 expression between CMV^–^EBV^–^ and single positive (CMV^–^EBV^+^ and CMV^+^EBV^–^) donors (Supplementary Figure 2C). In senescent cells, the defect in proliferative capacity is occasionally associated with telomere erosion. To test whether CD56^neg^ NK cells display telomere-dependent senescence, we measured telomere length by flow-cytometry based fluorescence in situ hybridization (FISH)-method. CD56^neg^ NK cells displayed intermediate telomere length compared to CD56^bright^ and CD56^dim^ NK cells, excluding critical telomere shortening in CD56^neg^ NK cells as an explanation for the low Ki-67 levels (Figure 2D). Interestingly, telomere shortening was associated with CMV and EBV co-infection in more immature cell subsets such as CD56^bright^ NK cells (Figure 2D) and naïve CD8^+^ T cells (Supplementary Figure 2D). Alternatively, cell senescence can be induced by DNA damage such as DNA double strand breaks (DSBs), which are marked by phosphorylation of histone 2A.X (γH2A.X) and that elicit DNA repair mechanisms collectively termed as DNA damage response (DDR). Similar to telomere-associated replicative senescence, stress-induced and age-associated senescence leads to the formation of DNA-damage foci and activation of the DDR [42]. To test for DDR activation in CD56^neg^ NK cells we first probed global phosphorylation of H2A.X and spontaneous activation of p38 mitogen-activated protein kinase (MAPK) [43,44] in CD56^neg^ and CD56^dim^ NK cells by FACS. Interestingly, H2A.X phosphorylation (Figure 2E, F) and p-p38 MAPK levels (Figure 2G) were lower in CD56^neg^ compared to CD56^dim^ NK cells (Figure 2G) and no significant effect of CMV and EBV co-infection was seen on H2A.X and p38 phosphorylation (Figure 2E-G). In a recent report from Hewitt et al., persistent DNA damage foci, that failed to be resolved by the DDR, mapped to telomeres/telomere associated structures rather than genomic DNA, presumably because of the inaccessibility of telomeres to the DDR machinery [42]. Such persistent DNA damage foci can be revealed by co-localization of γH2A.X with telomeres in a fluorescence *in situ* hybridization protocol and reliably identified senescent fibroblasts [42]. To further corroborate our findings of telomere-independent senescence, we applied this immune-FISH procedure on sorted CD56^neg^ and CD56^dim^ NK cells from all four subgroups of the cohort (n=3 each). Telomere-associated fluorescence (TAF) was defined as co-localization of γH2A.X with the telomere probe (Figure 2H). We found that the frequency of TAF-positive cells was strongly donor-dependent, ranging from 11 to 38% of NK cells, and it showed no clear association with CMV and EBV serostatus (Figure 2J and Supplementary Figure 2E, upper panels). There was no difference in the frequency of TAF-positive cells between CD56^neg^ and CD56^dim^ NK cells (18.2% vs 19.6% (mean TAF+ cells CD56^neg^ vs CD56^dim^). Likewise, the number of TAF foci / TAF+ cell was not different between the groups (Figure 2J and Supplementary Figure 2E, bottom panels). Taken together, our data argue against a senescent phenotype in CD56^neg^ NK cells.

### CD56^neg^ NK cells lack features of exhausted cells

**Figure 3:**
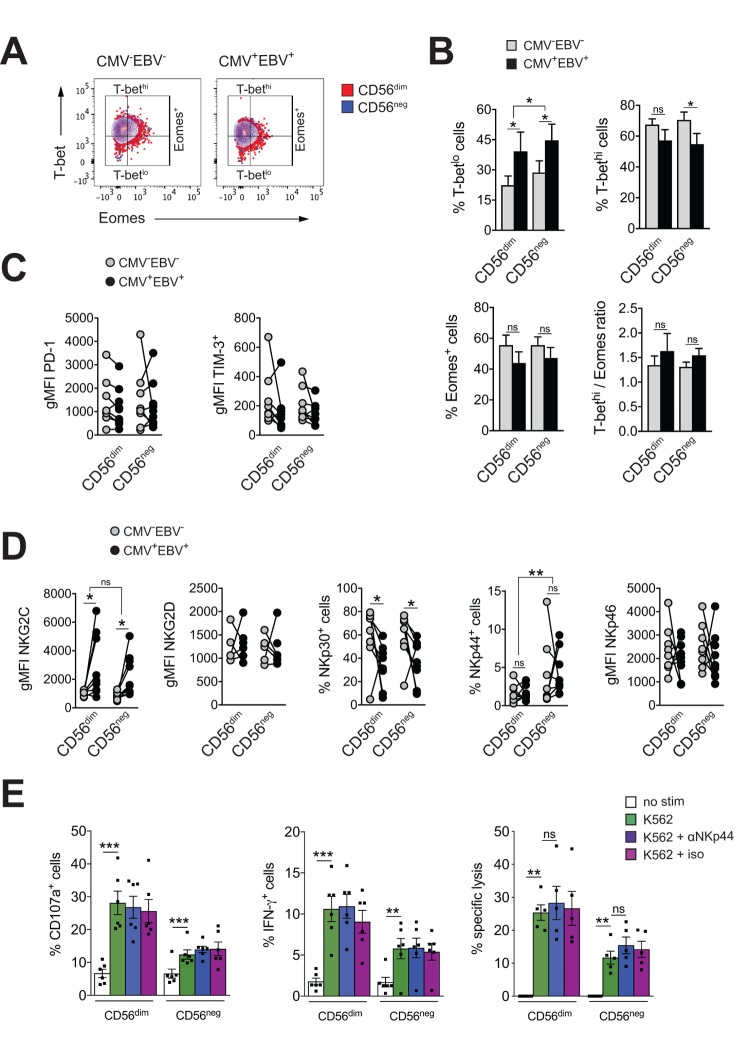
**CD56^neg^ NK cells lack features of exhausted cells.** (**A**) Overlay contour plot analysis comparing T-bet and Eomes expression in CD56^dim^ (red) and CD56^neg^ (blue) NK cells from a representative CMV^–^EBV^–^ (left panel) and CMV^+^EBV^+^ (right panel) donor. Gating strategy for T-bet-high (T-bet^hi^), T-bet-low (T-bet^lo^) and Eomes-positive (Eomes^+^) cells is indicated. (**B**) The percentage of T-bet^lo^, T-bet^hi^ and Eomes^+^ cells, as well as the T-bet / Eomes ratio (Tbet^hi^ / Eomes^+^) are shown in CD56^dim^ versus CD56^neg^ NK cells from CMV^–^EBV^–^ (gray bars, n=8/11) and CMV^+^EBV^+^ (black bars, n=8/14) donors analyzed as in (A). (**C**) PD1- and TIM-3 expression on CD56^dim^ and CD56^neg^ NK cells from CMV^–^EBV^–^ (gray circles, n=8/11) compared to CMV^+^EBV^+^ (black circles, n=9/14) donors. (**D**) Cell surface expression of activating NK cell receptors NKG2C and NKG2D and natural cytotoxicity receptors NKp30, NKp44 and NKp46 on CD56^dim^ and CD56^neg^ NK cells from CMV^–^EBV^–^ (gray circles, n=8/11) compared to CMV^+^EBV^+^ (black circles, n=9/14) donors. (**C, D**) Values are expressed as gMFI for unimodal data, and as % of positive cells for bimodal data. (**E**) FACS-sorted CD56^dim^ and CD56^neg^ NK cells were either left un-stimulated (empty bars), stimulated with K562 cells alone (green bars) or K562 cells and a blocking NKp44 monoclonal antibody (blue bars) or an isotype control (purple bars), respectively, and expression of CD107a, IFN-γ and target cell lysis was assessed as described. Experiments were performed on total PBMCs in (**A**-**D**) and on FACS-sorted CD56^dim^ and CD56^neg^ NK cells in (**E**). For parametric data mean ± SEM, for non-parametric data median ± IQR are shown. Data were analyzed by Student’s t-test and Mann-Whitney test, respectively. * p≤0.05, ** p≤0.005, *** p≤0.005, ns=not significant.

Exhausted NK cells have been described in chronic HIV, CMV and hepatitis B and C virus infection, and in human and animal cancers. Common characteristics of exhausted NK cells are impaired cytotoxicity and cytokine secretion, down-regulation of activating NK cell receptors and upregulation of inhibitory receptors, such as TIM-3 and PD-1. The transcription factors Eomesodermin (Eomes) and T-box transcription factor (T-bet) are modulated during maturation of NK cells, with progressive T-bet upregulation and Eomes down-regulation toward terminal differentiation [45]. Gill et al. described reduced expression of Eomes and T-bet as a molecular signature of exhausted NK cells in an animal model of lymphoma [46]. To test if expanding CD56^neg^ NK cells in CMV^+^EBV^+^ donors have features of exhausted cells, we first assessed expression of T-bet and Eomes in CD56^neg^ NK cells within all 4 subgroups of the cohort and compared them to CD56^dim^ NK cells. We found reduced T-bet expression in CMV^+^EBV^+^ individuals compared to CMV^–^EBV^–^ donors for both NK cell subsets (Figure 3A). This was exemplified by an increase in cells expressing low levels of T-bet (T-bet^lo^) and a decrease in cells with high T-bet expression (T-bet^hi^) (Figure 3B, upper panel). Eomes expression tended to be lower in CMV^+^EBV^+^ compared to CMV^–^EBV^–^ donors (Figure 3B, lower panel). CMV single positive (CMV^+^EBV^–^) donors showed a similar trend in T-bet and Eomes modulation as CMV^+^EBV^+^ donors (Supplementary Figure S3A), suggesting that changes in T-bet and Eomes expression are associated with CMV infection and are further enhanced by EBV co-infection. Nonetheless, differences in T-bet and Eomes expression between CD56^dim^ and CD56^neg^ NK cells were tenuous and cannot account for the reduced effector functions seen in CD56^neg^ NK cells.

Expression of the immune check-point inhibitors PD-1 and TIM-3 have been reported on NK cells in HIV and HCV infection as well as various tumor models, and blockade of each receptor alone, or in combination, was shown to reverse NK cell exhaustion [47–49]. We therefore tested for the expression of PD-1 and TIM-3 within all 4 subgroups of our cohort. There was no difference in PD-1and TIM-3 expression between CD56^dim^ and CD56^neg^ NK cells irrespective of the serostatus of the donor (Figure 3C). We next performed phenotypic analyses for the activating NK cell receptors NKG2C, NKG2D, and the natural cytotoxicity receptors (NCRs) NKp30, NKp44 and NKp46. The association of the activating NK cell receptor NKG2C with CMV is well established. Correspondingly, we found increased levels of NKG2C on both NK cell subsets with CMV infection that were further increased by EBV co-infection (Figure 3D and Supplementary Figure S3B). There was no significant difference in NKG2C expression between CD56^dim^ and CD56^neg^ NK cells (Figure 3D and Supplementary Figure S3B). In contrast, expression of the natural cytotoxicity receptor NKp30 was reduced with CMV and EBV co-infection on both NK cell subsets (Figure 3D and Supplementary Figure S3B). Further, CD56^neg^ NK cells had higher levels of NKp44 expression than CD56^dim^ NK cells irrespective of the serostatus of the donors (Figure 3D and Supplementary Figure S3B). In circulation, NKp44 is found on activated NK cells only, and engagement of the receptor by activating ligands mediates release of cytotoxic granules, IFN-γ and TNF-α. In contrast to other NCRs, NKp44 is endowed with an inhibitory function as well, and it has been postulated that tumors exploit this axis to escape NK cell attack [50,51]. Therefore, we next blocked NKp44 with a monoclonal antibody on sorted CD56^dim^ and CD56^neg^ NK cells, or an isotype control, respectively, and tested expression of CD107a, and IFN-γ production in response to K562 target cells as well as lysis of K562 target cells. While both CD56^dim^ and CD56^neg^ NK cells readily increased CD107a and IFN-γ expression after target cell stimulation, no effect on effector functions was seen with NKp44 blockade or the isotype control (Figure 3E) arguing against a role for NKp44 in inhibiting NK cell effector functions.

## CONCLUSIONS

In a cohort of healthy donors >60 years of age, we show that CD56^neg^ NK cells expanded in CMV / EBV co-infection and that their frequency correlated with the immune risk profile (IRP). CD56^neg^ NK cells were less functional when compared to CD56^dim^ cells of the same donor in terms of their degranulation, killing capacity and IFN-γ production when stimulated with K562 target cells or IL-12 / IL-18, a feature that was more pronounced in CD56^neg^ NK cells from CMV^+^EBV^+^ donors. Phenotypically CD56^neg^ NK cells were mature cells, yet compared to CD56^dim^ NK cells they were characterized by a CD57^low^KIR^low^ phenotype, reduced T-bet expression and had longer telomeres compared to CD56^dim^ NK cells. CD56^neg^ NK cells thus distinguished themselves from CD56^dim^ cells, the main effector population, as a distinct cell subset. Neither reduced expression of activating NK cell receptors, nor increased expression of PD-1 and TIM-3 accounted for reduced functionality of CD56^neg^ NK cells. Likewise, no cell senescence characteristics were detected in this cell subset. In all, our data suggest that CD56^neg^ NK cells can be viewed as an additional marker of immune risk in the aging host, and that EBV has a previously unrecognized role in immune senescence.

## MATERIALS AND METHODS

### Blood sample collection and assessment of donor CMV and EBV serostatus

The cohort of elderly donors (>60 years (median 64 years, range 62-70 years)) was recruited at the University Hospital Basel, Blood Transfusion Centre of both Basel, Switzerland. After written informed consent was obtained, healthy blood donors aged >60 years who routinely presented at the center were assessed for their serological EBV status by multiplex microparticle technology (Luminex 200 Technology, Luminex, Austin, TX, USA). Specifically, we probed for IgG antibodies specific for the EBV antigens VCA, EBNA-1 and EA (EBV-IgG Plus Test, AtheNA Multi-Lyte, Inverness Medical, Princeton, NJ, USA). We recruited 17 EBV-negative individuals and 38 EBV-positive age- and sex-matched controls for a second blood donation where buffy coats were obtained. At the recall blood donation, EBV serology was repeated for all EBV-negative donors with multiplex microparticle technology. CMV serostatus was recorded from previous donations for CMV-positive donors or assessed in serum or plasma at the time of the recall donation (if previously not tested or CMV-negative) utilizing CMV lysate-coated microparticles (strain AD169) for the capture of human anti-CMV IgG (ARCHITECT CMV IgG Assay, Abbott, Baar, Switzerland). Samples from this cohort were used for all experiments of this study except in Figure 1A, left panel, where the young control cohort was used.

20 young donors (<35 years (median 31 years, range 25-34 years)) who were recruited at the University College London after approval of the Local Research Ethics Committee of the Royal Free and the University College London Medical School, served as a control cohort. After informed consent was obtained, whole blood was collected in standard heparinized tubes and PBMCs were isolated using Ficoll Histopaque (Amersham Biosciences). CMV status was obtained as described in [52]. Briefly, PBMC were stimulated with CMV viral lysate overnight and CMV status was obtained by flowcytometry-based assessment of IFN-γ production in CD4^+^ T cells. Previous data from our group had shown good concordance between IFN-γ responses and CMV IgG serology as obtained from the diagnostic laboratory of University College London [52].

### Cell isolation and sorting of NK cells

PBMCs were isolated from buffy coat preparations by standard Lymphoprep (STEMCELL Biotechnology) gradient centrifugation and stored in fetal calf serum (FCS) 10% DMSO in liquid nitrogen. NK cells were pre-sorted from frozen PBMCs samples by magnetic bead isolation using the NK Cell Isolation Kit (MACS Technology, Miltenyi Biotec) or the NK Cell Enrichment Kit (EasySepTM from STEMCELL Biotechnology). Enriched NK cells were then stained with anti-CD3 (UCHT1), anti-CD7 (M-T701), anti-CD56 (HCD56), and anti-CD16 (3G8) (all from BioLegend) and CD56^dim^ and CD56^neg^ NK cells were sorted on a FACSAria (BD Biosciences) cell sorter. Sorted cells were cultured in *complete medium* (RPMI 1640 supplemented with 10% heat-inactivated FCS, 100 U/ml Penicillin, 100 mg/ml Streptomycin, and 2 mM L-glutamine; all from Invitrogen) at 37°C for 2h prior to the functional assays. Sorted NKs were used in Figure 1D, 1E, 1F; Figure 2H, 2J; Figure 3E; Supplementary Figure S2E. All other experiments were performed on bulk PBMCs.

### Flow cytometry

The following antibodies (all from BioLegend unless otherwise indicated) were used: anti-CD3 (OKT3), anti-CD56 (HCD56), anti-CD16 (3G8), anti-CD7 (M-T701), anti-CD4 (A161A1), anti-CD8 (RPA-T8), anti-CD27 (LG.3A10), anti-CD28 (CD28.A), anti-CD45RA (HI100), anti-TIM3 (F38-2F2), anti-PD-1 (EH12.2H7), anti-CD158e1 (DX9), anti-CD158b (DX27), anti-CD158f (URR 1), CD158 (HP-MA4) anti-T-bet (4B10), anti-Eomes (WD1928, EBioscience), anti-NKG2A (131411, R&D Systems), anti-NKG2C (134591, R&D Systems), anti NKG2D (149810, R&D Systems), anti-NKp30 (Z25, Beckman Coulter Diagnostics), anti-NKp44 (44.189, eBioscience), anti-NKp46 (9E2), Ki67 (B56, BD Biosciences). A fixable live – dead cell stain (UV Zombie, BioLegend) was used to exclude dead cells throughout. Biotin-conjugated antibodies were detected using Cy5- or Cy3-conjugated Streptavidin (BioLegend). T-bet, Eomes and Ki-67 staining was performed with the Foxp3 Staining Set (Miltenyi Biotec) according to the manufacturer’s instructions. All samples were acquired on a LSRFortessa flow cytometer (BD Biosciences). Data were analyzed using FlowJo®_V10.4 software (Tree Star, Ashland, OR).

### Phosphoflow cytometry

After cell surface staining as defined above cells were fixed with PBS 2% paraformaldehyde (PFA) for 10min at 37°C and permeabilized with ice-cold Perm Buffer III (BD Biosciences). Cells were either stored in Perm Buffer III at -20°C or stained immediately with the following antibodies for 30 min at room temperature: anti-γH2A.X-APC (20E3, BioLegend), anti-p-p38 MAPK (pT180/pY182)-PE (36/p38, BD Biosciences). Samples were acquired immediately after the staining on a LSRFortessa flow cytometer (BD Biosciences). Data were analyzed using FlowJo®_V10.4 software (Tree Star, Ashland, OR).

### Multi-color flow-FISH analysis of telomere length

Relative telomere length of NK and T cell subsets was assessed as previously described [53]. Briefly, PBMCs were first stained with a biotinylated anti-CD28 (CD28.A) antibody, followed by Streptavidin-conjugated-Cy3, a fixable live – dead cell stain (UV Zombie, BioLegend) and anti-CD56 (HCD56), anti-CD7 (M-T701), anti-CD3 (OKT3), anti-CD4 (A161A1), anti-CD8 (RPA-T8), anti-CD16 (3G8), anti-CD45RA (HI100), anti-CD27 (LG.3A10). Samples were then washed in PBS, fixed with 1 mM BS3 (Thermo Scientific UK) and quenched with 50 mM Tris–HCl in PBS (pH 7 2, 20 min, room temperature). For the hybridization step, cells were resuspended in 70% deionized formamide, 2.85 mM Tris–HCl pH 7.2, 1.4% BSA and 0.2 M NaCl and 0.75 μg/ml of PNA TelC-Cy5 probe (PNA Bio, US) was added. Samples were then heated for 10 min at 82°C before being rapidly cooled down on ice. After 1 hour of incubation at room temperature, samples were washed twice in 70% deionized formamide, 14.25 mM Tris–HCl pH 7.2, 0.14% BSA, 0.2 M NaCl, 0.14% Tween-20 in 2% BSA/PBS twice before acquisition on a LSRFortessa cytometer (BD Biosciences). Quantum Cy5 molecules of Equivalent Soluble Fluorochrome (MESF) beads (Bangs Laboratories, USA) were acquired alongside samples in each experiment to ensure standardization of FACS machine set up. Data were analyzed using FlowJo®_V10.4 software (Tree Star, Ashland, OR).

### CD107a and IFN-γ assay in sorted NK cells

Flow-sorted CD56^dim^ and CD56^neg^ NK cells were cultured in *complete medium* at 37°C for 2 hours prior to stimulation. 100’000 NK cells / well were seeded in 96-well U-bottom plates and activated either with IL-12 (10 ng / μl) and IL-18 (50 ng / μl) or with K562 target cells at an effector-to-target ratio of 5:1 in *complete medium*. Brefeldin A, Monensin and an anti-CD107a antibody (BD Biosciences) were added after 30 minutes of culture. NK cells were then harvested after 6 hours of activation / co-culture and fixed in Fixation / Permeabilization solution (BD Bioscience) for 15 minutes at room temperature. For the detection of intracellular IFN-γ, samples were stained with an anti-IFN-γ antibody (B27, Immunotools) in Perm/Wash buffer (BD Biosciences) for 30 min at room temperature. All samples were acquired immediately after the staining on a LSRFortessa flow cytometer (BD Biosciences). Data were analyzed using FlowJo®_V10.4 software (Tree Star, Ashland, OR).

### Cytotoxicity assay

Flow-sorted CD56^dim^ and CD56^neg^ NK cells were cultured in complete medium at 37°C for 2 hours prior to stimulation. 100’000 NK cells were seeded in 96-well U-bottom plates and stimulated with K562 target cells labeled with Cell Proliferation Dye eFluor670 (ThermoFisher) at an effector-to-target ratio of 5:1 in *complete medium*. To control for spontaneous cell death, labeled target cells were plated in the absence of effector cells. After 4 hours of incubation, cells were stained with Zombie Green viability dye (BioLegend) and analyzed by FACS. % specific lysis was calculated as (% dead target cells in experimental condition - % dead target cells in control) / (100% - % dead target cells in control) x 100.

### Telomere fluorescence in situ hybridization

Flow-sorted CD56^dim^ and CD56^neg^ NK cells were cytocentrifuged onto poly-L-lysine coated glass slides (Cytospin, Thermo Scientific). Staining for telomere-associated γH2A.X foci (TAF) was then performed as previously described [42]. Slides were air dried prior to hybridisation with 40 pM PNA probe targeting the TelC telomeric repeat (Panagene, TelC Cy3, #14 1224PL-01) for 2 hours. Sections were then counter stained for γH2A.X (Ser139, Cell Signaling #9718), followed by incubation with biotinylated secondary antibody (BA-1000, Vector) and FITC-streptavidin (A-2011). Slides were subsequently washed in formamide/SSC buffer prior to mounting with Vectorshield/DAPI (Vector Laboratories). Imaging was performed using a Leica SPE2 confocal microscope (Leica Microsystem). Analysis was performed using Fiji image analysis software (Fiji.sc).

### Statistics

GraphPad Prism software was used to perform all statistical analyses. For parametric data Student’s t-test or repeated measures ANOVA test with Greenhouse-Geisser correction were used. For non-parametric data Wilcoxon matched-pairs signed rank test or Friedman test were used. P values <0.05 were considered significant.

## Supplementary Material

Supplementary Figure 1

Supplementary Figure 2

Supplementary Figure 3
