# Supplementary material for: CD56-negative NK cells with impaired effector function expand in CMV and EBV co-infected healthy donors with age
Source: Aging (Albany NY). 2019 Jan 27;11(2):724–40. doi: 10.18632/aging.101774 (PMC6366961; doi:10.18632/aging.101774)
Supplement: Supplementary Figure 1 [file aging-11-101774-s001.pdf]

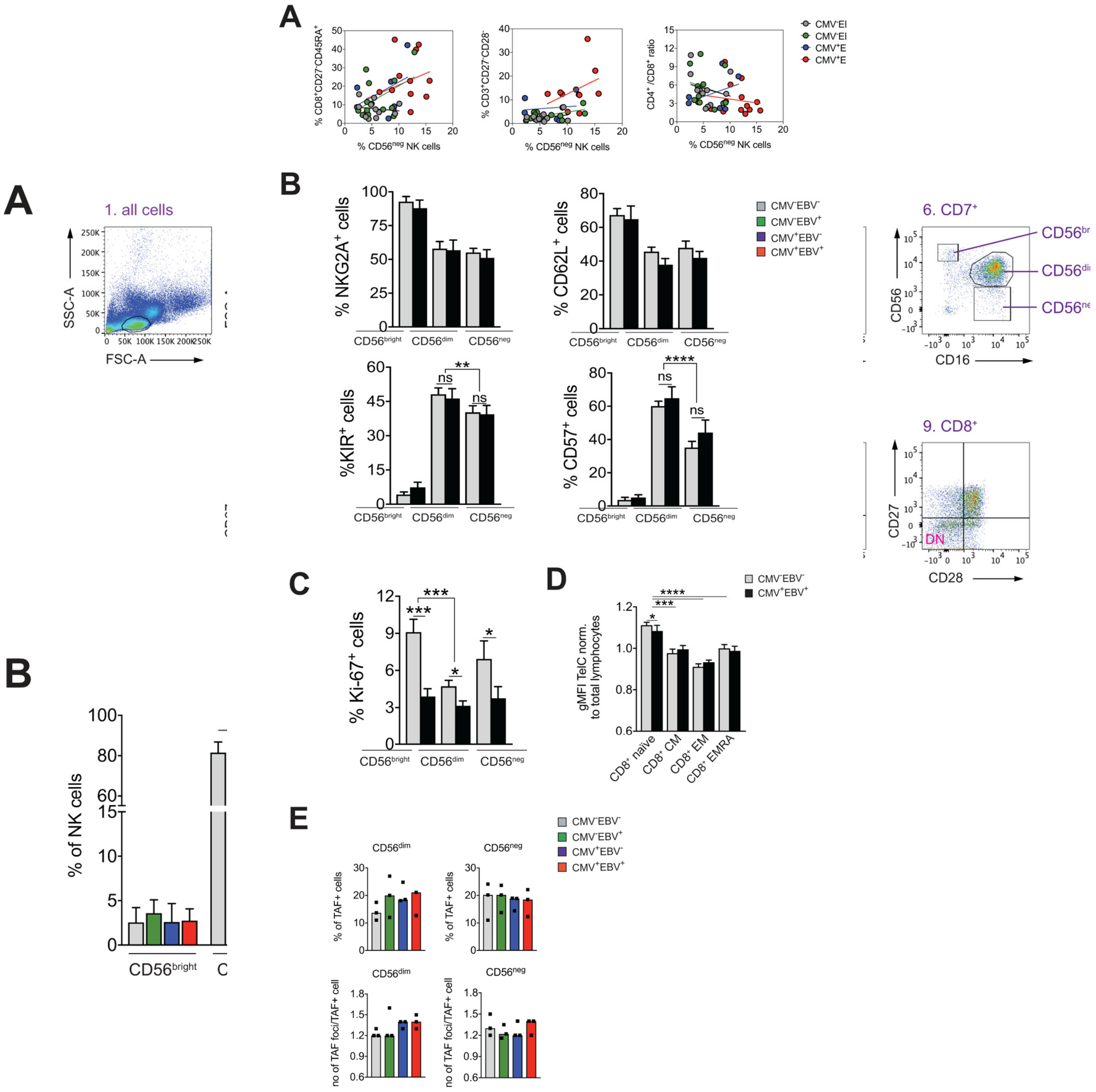

Figure S1. Experimental gating strategy for NK and T cell subsets and frequencies of NK cell subsets within cohort subgroups. (**A**) For all FACS experiments performed on total PBMCs, the following gating strategy was applied: (**1**) Lymphocytes were identified by sideward (SSC) and forward scatter (FSC) parameters. (**2**) Doublet discrimination gating was performed by plotting FSC-area (FSC-A) versus FSC-height (FSC-H) and excluding cells with disproportionate cell size as indicated. (**3**) Dead cells were excluded by staining with a fixable viability dye. Only living cells were used for further analysis. For analysis of NK cells, we first excluded T cells by gating on $CD3^{-}$ lymphocytes only (**4**), followed by exclusion of $CD7^{-}$ cells (**5**). These cells were then used to define $CD56^{bright}$, $CD56^{dim}$ and $CD56^{neg}$ NK cells, as indicated in (**6**) T cells were identified by gating on $CD3^{+}$, $CD56^{-}$ cells (**4**) followed by CD4 and CD8 gating (**7**). $CD4^{+}$ and $CD8^{+}$ T cells were further divided into naïve (N), central memory (CM), effector memory (EM) and terminally-differentiated effector memory (EMRA) subsets, based on their expression of CD27 and CD45RA (**8** and **10**). Double-negative (DN) $CD4^{+}$ and $CD8^{+}$ T cells were determined by CD27 and CD28 gating (**9** and **11**). (**B**) Frequencies of $CD56^{bright}$, $CD56^{dim}$ and $CD56^{neg}$ NK cells – as determined by FACS analysis in total PBMCs– are shown in a cohort of HDs >60 years of age stratified as $CMV^{-}EBV^{-}$ (n=11/11), $CMV^{-}EBV^{+}$ (n=21/24), $CMV^{+}EBV^{-}$ (n=6/6), and $CMV^{+}EBV^{+}$ (n=12/14). * p≤0.05, ** p≤0.005, *** p≤0.005, **** p≤0.0005.
