# Supplementary material for: CD56-negative NK cells with impaired effector function expand in CMV and EBV co-infected healthy donors with age
Source: Aging (Albany NY). 2019 Jan 27;11(2):724–40. doi: 10.18632/aging.101774 (PMC6366961; doi:10.18632/aging.101774)
Supplement: Supplementary Figure 2 [file aging-11-101774-s002.pdf]

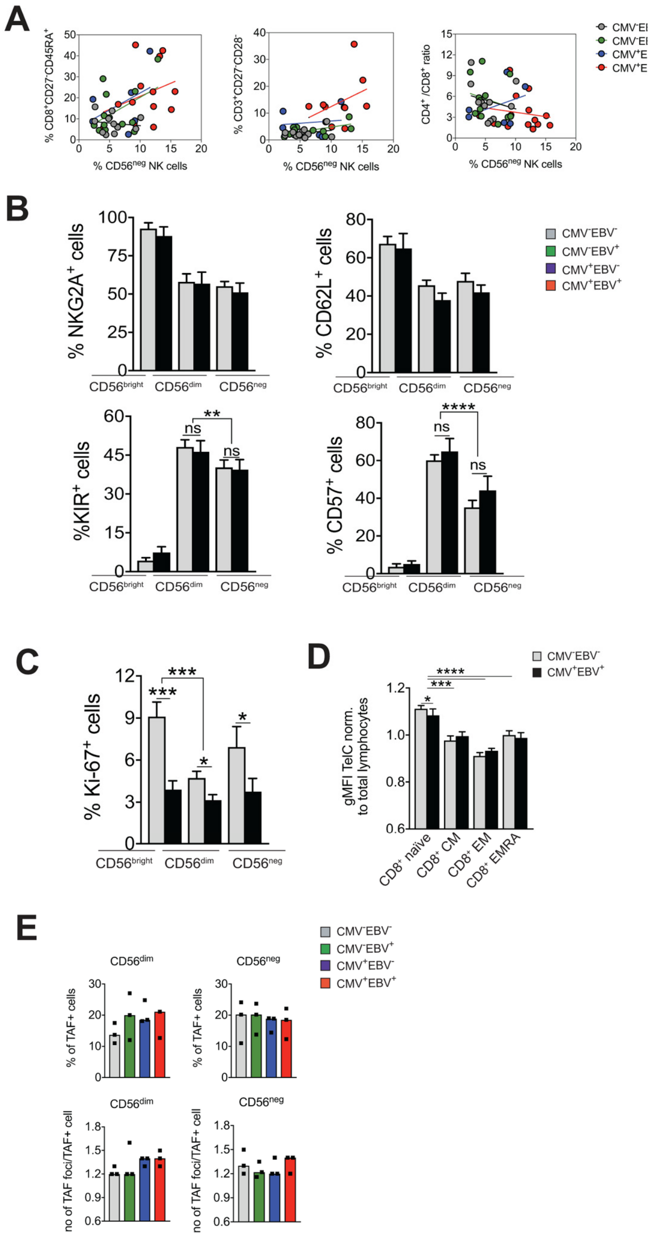

Figure S2. Impact of individual CMV and EBV infection on the correlation of $CD56^{neg}$ NK cells with the IRP, the differentiation stage and cell senescence characteristics. (**A**) Frequencies of $CD56^{neg}$ NK cells in relation to $CD8^+$ EMRA T cells (left panel), $CD27^-CD28^-$ T cells (middle panel) and the CD4 / CD8 T cell ratio (right panel) in CMV^-EBV^- (gray dots, n=11/11), CMV^-EBV^+ (green dots, n=13/24), CMV^+EBV^- (blue dots, n=6/6) and CMV^+EBV^+ (red dots, n=13/14) donors as assessed by FACS analysis in total PBMCs. No significant correlation was found for any subgroup as analyzed by linear regression analysis. (**B**) The differentiation stage of $CD56^{bright}$, $CD56^{dim}$ and $CD56^{neg}$ NK cells in CMV^-EBV^- (gray bars, n=11/11), CMV^-EBV^+ (green bars, n=13/24), CMV^+EBV^- (blue bars, n=6/6) and CMV^+EBV^+ (red bars, n=13/14) donors was assessed by FACS staining for cell surface expression of NKG2A, CD62L, KIR and CD57 in total PBMCs. No significant differences in NKG2A, CD62L, KIR and CD57 expression were found in single positive compared to CMV^-EBV^- donors. (**C**) Proliferation was assessed directly *ex vivo* by FACS analysis for Ki-67 expression in CMV^-EBV^- (gray bars, n=10/11), CMV^-EBV^+ (green bars, n=9/24), CMV^+EBV^- (blue bars, n=6/6) and CMV^+EBV^+ (red bars, n=11/14) donors. (**D**) Telomere length in $CD8^+$ T cell subsets as assessed by FACS-based FISH-technique is shown for CMV^-EBV^- (gray bars, n=10/11) and CMV^+EBV^+ (black bars, n=10/14) donors. Data are shown as geometric mean of fluorescence intensity (gMFI) of the telomere probe (TelC), normalized to the gMFI TelC value of the total lymphocyte population for each donor. (**E**) Telomere fluorescence in situ hybridization (TAF) was analyzed in all 4 subgroups of the cohort (n=3 each). Top panels show the frequency of TAF+ cells, bottom panels the number of TAF / TAF+ cell in $CD56^{dim}$ and $CD56^{neg}$ NK cells. (**A-D**) Experiments were performed on total PBMCs. (**E**) Experiments were performed on FACS-sorted $CD56^{dim}$ and $CD56^{neg}$ NK cells. For parametric data mean ± SEM, for non-parametric data median ± IQR are shown throughout. Data were analyzed by Student's t-test and Mann-Whitney test, respectively. * p≤0.05, ** p≤0.005, *** p≤0.005, **** p≤0.0005, ns=not significant.
