# Supplementary material for: CD56-negative NK cells with impaired effector function expand in CMV and EBV co-infected healthy donors with age
Source: Aging (Albany NY). 2019 Jan 27;11(2):724–40. doi: 10.18632/aging.101774 (PMC6366961; doi:10.18632/aging.101774)
Supplement: Supplementary Figure 3 [file aging-11-101774-s003.pdf]

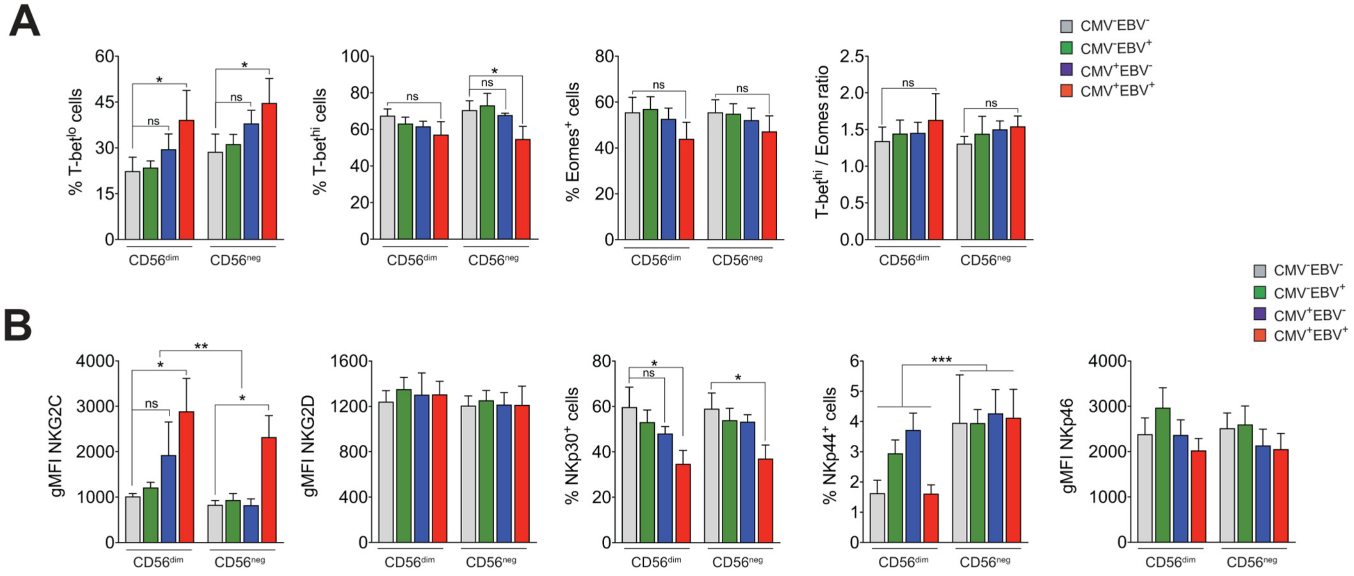

Figure S3. Expression of T-bet and Eomes and activating NK cell receptors in relation to CMV and EBV infection. (A) The frequency of T-bet^{lo}, T-bet^{hi} and Eomes^{+} cells, as well as the T-bet / Eomes ratio (Tbet^{hi} / Eomes^{+}) are shown in CMV^{-}EBV^{-} (gray bars, n=11/12), CMV^{-}EBV^{+} (green bars, n=9/24), CMV^{+}EBV^{-} (blue bars, n=6/6) and CMV^{+}EBV^{+} (red bars, n=11/14) donors within CD56^{dim} and CD56^{neg} NK cell subsets. To test for an effect of CMV and EBV infection we compared T-bet and Eomes expression of each cohort subgroup and within each cell subset. (**B**) Cell surface expression of activating NK cell receptors NKG2C and NKG2D and natural cytotoxicity receptors NKp30, NKp44 and NKp46 in CMV^{-}EBV^{-} (gray bars, n=11/11), CMV^{-}EBV^{+} (green bars, n=9/24), CMV^{+}EBV^{-} (blue bars, n=6/6) and CMV^{+}EBV^{+} (red bars, n=11/14) donors within CD56^{dim} and CD56^{neg} NK cell subsets. To test for an effect of CMV and EBV infection we compared expression of activating NK cell receptors and NCRs within each cohort subgroup and for each cell subset. All experiments were performed on total PBMCs. For parametric data mean ± SEM, for non-parametric data median ± IQR are shown. Data were analyzed by Student's t-test and Mann-Whitney test, respectively. * p≤0.05, ** p≤0.005, *** p≤0.005, ns=not significant.
